# Osseointegration of TI6Al4V dental implants modified by thermal oxidation in osteoporotic rabbits

**DOI:** 10.1186/s40729-016-0051-5

**Published:** 2016-07-21

**Authors:** Oscar G. Bodelón, Celia Clemente, Miguel Angel Alobera, Soledad Aguado-Henche, María Lorenza Escudero, María Cristina García Alonso

**Affiliations:** 1 Department of Characterization, Quality and Safety, Institute of Food Science, Technology and Nutrition (ICTAN-CSIC), Madrid, Spain; 2 Department of Human Anatomy and Embryology, School of Medicine, University of Alcalá, Alcalá de Henares, Spain; 3 Stomatology Department IV, Complutense University of Madrid (UCM), Madrid, Spain; 4 Department of Surface Engineering, Corrosion and Durability, National Center for Metallurgical Research (CENIM), CSIC, Avda. Gregorio del Amo 8, 28040 Madrid, Spain

**Keywords:** Growth hormone, Histomorphometry, In vivo, Oxidation treatment, Ti6Al4V

## Abstract

**Background:**

In this work, the effect of the heat treatment on Ti6Al4V implants and topical administration of growth hormone to address a better osseointegration in osteoporotic patients has been analysed.

**Methods:**

The osseointegration process of Ti6Al4V implants modified by oxidation treatment at 700 °C for 1 h and the influence of local administration of growth hormone (GH) in osteoporotic female rabbits after 15 and 30 days of implantation have been studied. Bone response was analysed through densitometric and histomorphometric studies. Characterization of the surface was provided by scanning electron microscopy.

**Results:**

The oxidation treatment promotes the formation of an oxide scale grown on the Ti6Al4V implants that alters the nanoroughness of the surface. Bone mineral density (BMD) increases from 0.347 ± 0.014 (commercial) to 0.383 ± 0.012 g cm−2 (modified), and bone-to-implant contact (BIC) goes from 48.01 ± 14.78 (commercial) to 55.37 ± 15.31 (modified) after 30 days of implantation.

**Conclusions:**

The oxidation treatment on the Ti6Al4V dental implants enhances the early bone formation at the longest periods of implantation. No significant differences in the BMD and BIC results in healthy and osteoporotic rabbits were revealed with respect to the local administration of GH in the implantation site.

## Background

The metallic biomaterials used in the substitution of hard tissues are subjected to the action of the physiological environment and mechanical efforts like fatigue, wear or friction that alter the operation success of implants and affect drastically the electrochemical properties of the surface. That is the case of Ti and its alloys, in which in vivo conditions alter the stability of the passive layer and provoke the release of both metallic ions and particles [1].

The search for new treatments that increase the wear resistance, improve the bioactivity in order to facilitate the formation of bone tissue around it and decrease the titanium release is increasingly important. Among them, thermal oxidation treatments aimed to obtain “in situ” ceramic coatings can offer thick, highly crystalline oxide films with very good protective performances [2, 3]. The authors have proved that oxidation treatments of Ti6Al4V alloy at 700 °C for 1 h provoke the formation of an oxide layer, mainly composed of rutile [4] whose ion release is reduced to the half [5]. In vitro experiments with primary osteoblasts cell culture revealed that the surface modification does not alter even improve the excellent biocompatible behaviour. In fact, cell adhesion is favoured on the thermally treated surfaces [6]. However, in vivo evidences have not been previously studied by the authors. At the same way, sandblasting of Ti6Al4V alloys in order to increase the roughness and subsequent thermal treatment improves the osteoblast response. The enhancement of the osseointegration process can be achieved modifying the quality of the surface of the implant in terms of chemical, physical and topographical properties, all of them influencing the functional activity of cells around the implant surface.

On the other hand, in those cases where there is a delay in the physiological mechanisms of bone repair, either by aging or due to problems of osteoporosis, the solution must be taken by means of external substances that stimulate the bone metabolism. Patients diagnosed with systemic diseases such as osteoporosis and diabetes are considered to be medically compromised for implant therapy. Research has been focused on the local application of substances on the implant surface [7–9], or directly in the implantation site, able to accelerate the osseointegration process. Hormones [10–12], growth factors [13, 14] and osseoconductive proteins [15] are being used to stimulate the bone growth. Studies carried out by Becker et al. [16] revealed that local application of diverse growth factors (IGF-I and PDGF) induce the statistical increase in bone repair and the increase of bone density, around Ti implants, compared with the control group. Numerous studies highlight the importance of the growth hormone (GH) in the repair of bone fractures as young as old animals. The administration is able to increase up to 400 % the mechanical properties with respect to the control group [17, 18], stimulate the osteoblast activity and enhance the bone neoformationa around implants [14].

The aim of this study is to study the influence of the thermal treatment of Ti6Al4V dental implants and topical administration of growth hormone on the osseointegration process of osteoporotic rabbits during the first month of implantation.

## Methods

### Implant preparation and characterization

Threaded commercial titanium alloy implants (3.3 mm diameter and 8 mm length) from Zimmer® were used as control implants. They consisted of Ti6Al4V screws blasted with hydroxyapatite and subsequent acid attack with HCl. Screws with these prior surface treatments were submitted to a thermal treatment. The oxidation treatment was performed at 700 °C for 1 h, in air. After oxidation, samples were removed from the furnace and cooled at room temperature. Commercial and heat-modified screws were gamma-ray sterilized prior to implantation.

Scanning electron microscope (SEM) was used to characterize the surface morphology of the Ti implants. The surfaces were analysed by using a JEOL-6500F microscope equipped with a field emission gun (FEG) coupled with an energy dispersive X-ray (EDX) spectrometer. The images were taken using secondary electrons.

### Animals and treatment

The experimental protocol of this study and animal care conformed to the European Communities Council Directive of 24 November 1986 (86/609/EEC) [19] and has been independently reviewed and approved by the Madrid Community Ethics Committee for Regional Clinical Research (CEIC-R). Forty New Zealand female rabbits of approximately 5 kg and 11 months old were chosen as experimentation animals.

Animals were divided into healthy non-ovariectomised group and ovariectomised (OVX) group to carry out the tests. In order to induce osteoporosis, female rabbits underwent bilateral ovariectomy under general anaesthesia and were fed a hypocalcic diet (8.2 g kg^−1^ calcium and 6 g kg^−1^ phosphorous) for 8 weeks [20]. Diet mainly consisted of 74.2 % barley, 20 % wheat bran, 5 % soy and 0.3 % salt was supplied by PanLab SL (Harvard Bioscience Group, Barcelona, Spain). This procedure was selected because the treatment based only on the ovariectomy and hypocalcic diet provokes spontaneous fractures [21, 22].

Eight weeks post-surgery, rabbits were subjected to a densitometry to the vertebral column and tibia bone to verify the establishment of osteoporosis model [21]. For the densitometric analysis, a Norland XR-26 densitometer was used (Norland Co., Fort Atkinson, WI, USA) calibrated prior to the measurement. The exploration parameters were as follows: speed 40 mm s^−1^, resolution 1.0 × 1.0 mm, and measurement resolution 0.5 × 0.5 mm. Forty percent of the total length of each bone was analyzed, including metaphysis and diaphysis regions to verify differences between bone mass of healthy and osteoporotic groups. The value obtained was the bone mineral density (BMD) in grammes per centimetre.

### Implant procedure. Surgery

**Fig. 1:**
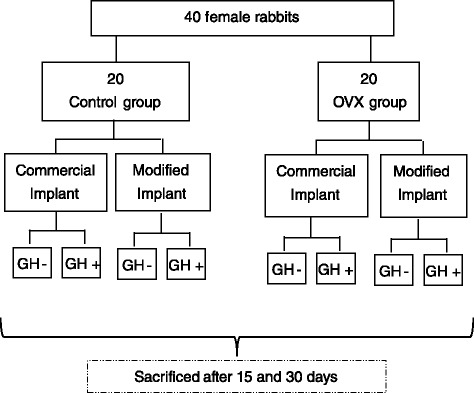
Schematic diagram of the classification of experimental animals in groups

The experimental design carried out with the experimental animals is summarized in Fig. 1. Rabbits were randomly divided into two groups: control (healthy rabbits) and OVX (osteoporotic rabbits). Both of them were subjected to surgery to insert commercial (no thermally treated Ti6Al4V) and modified (thermally treated Ti6Al4V) implants. The half of the healthy and osteoporotic rabbits was treated with 4 IU of recombinant human growth hormone (rhGH) as lyophilized powder (Genotonorm® Pfizer, NY, USA) directly located in the place of insertion. Healthy and osteoporotic rabbits without implants were also included as reference at both implantation times in the study.

**Fig. 2:**
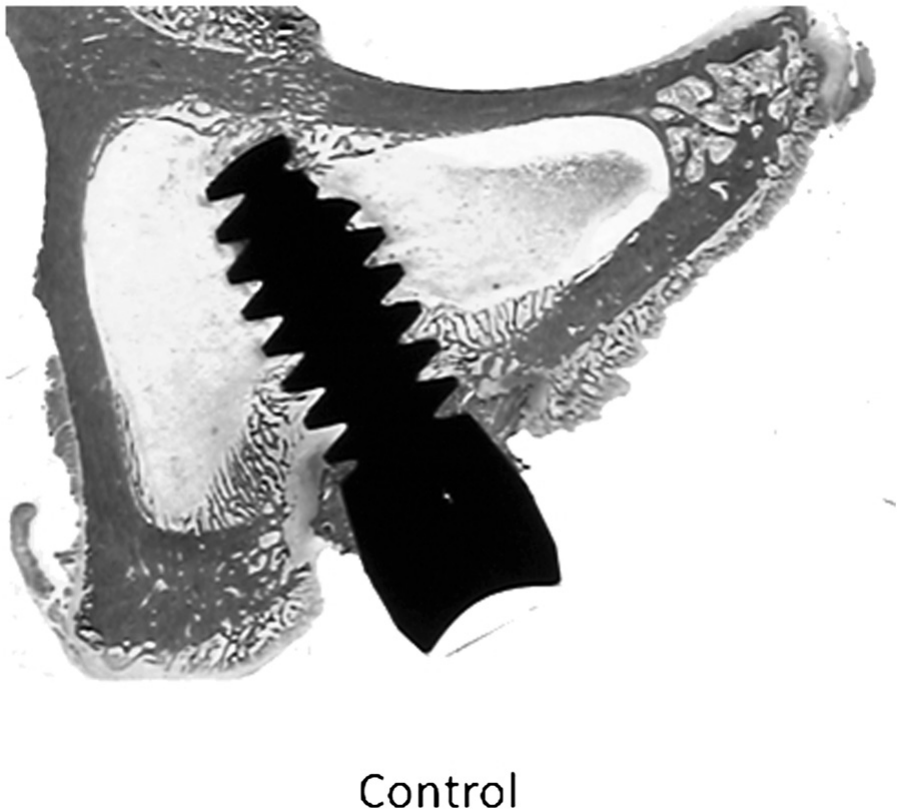
Transcortical osteotomy with Ti6Al4V implant inserted in the tibia bone

Incision was performed in the inner side of the proximal epiphysis of each tibia, under intramuscular anaesthesia. Transcortical osteotomy followed by drilling to generate a bed of 3.1 mm diameter and 8 mm deep was made, where the implant was inserted until touching the opposite cortical bone (Fig. 2). In the experimental group with local GH, 4 IU of rhGH as powder was added into the bone hole just before the insertion of the Ti6Al4V implant. Commercial implant in the right tibia and the modified implant in the left tibia were inserted. Only one implant was inserted in each tibia.

The injury was sutured with absorbable material after the implantation. Antibiotics and anti-inflammatory agents were delivered in the postoperative period to prevent infection and pain. Rabbits were sacrificed after 15 and 30 days of implantation by intravenous injection of 0.4 mg sodium pentobarbital (Dolethal®, Vetoquinol, Cedex, Francia) diluted in serum (Fig. 1).

Tibiae were cleaned from soft tissues to determine the bone mineral density (BMD). Densitometries at 0.5 mm above and 0.5 mm below the Ti6Al4V implant were acquired to compare the BMD of control and OVX groups.

Histological study of each tibia (including the Ti6Al4V implant) for control and modified implants and with and without GH was carried out. Clean hard tissues were fixed in 10 % pH 7 buffered formaldehyde and dehydrated in grading alcohol concentrations. Tibiae were cut into blocks and histologically prepared according to the modified Donath and Breuner method [23]. The preparation of hard tissue consisted of embedding in light-polymerizing 2-hydroxietyl-metacrylate. These blocks were cutting in five sections of about 200 μm and then grinding until achieving cross-sections of about 80-μm final thicknesses with an EXAKT cutting and grinding equipment (EXAKT, Norderstedt, Germany).

Bone-implant interface sections were examined under the optical microscope (Zeiss, Oberkpchen, Germany) using histological laboratory stains such as toluidine blue with Weigert haematoxylin (Merck, Kenilworth, NJ, USA) that allows the differentiation between osteoblasts and osteoclasts [24].

The images were processed and cross-sections were compared by means of the MIP-4 image analyser software (Digital Image Systems, SL, Barcelona) in order to quantify the bone fraction. The MIP-4 software is able to perform area and volume measurements through a computerized system connected to the optical microscope and histological lens. Area and length measurements on the images captured from the microscope were attained. All images were processed with ×10 magnification objective. The bone-to-implant contact (BIC) is calculated as the ratio of the length of the implant in contact to bone tissue and the implant perimeter, i.e. the percentage of the implant surface in contact with bone.

The quantitative results were processed with the statistical package Statgraphics plus 5.1. The significance of the differences between the groups was studied according to Student’s *t* test and the one-way ANOVA test (analysis of variance). *p* value was 0.05.

## Results

**Fig. 3:**
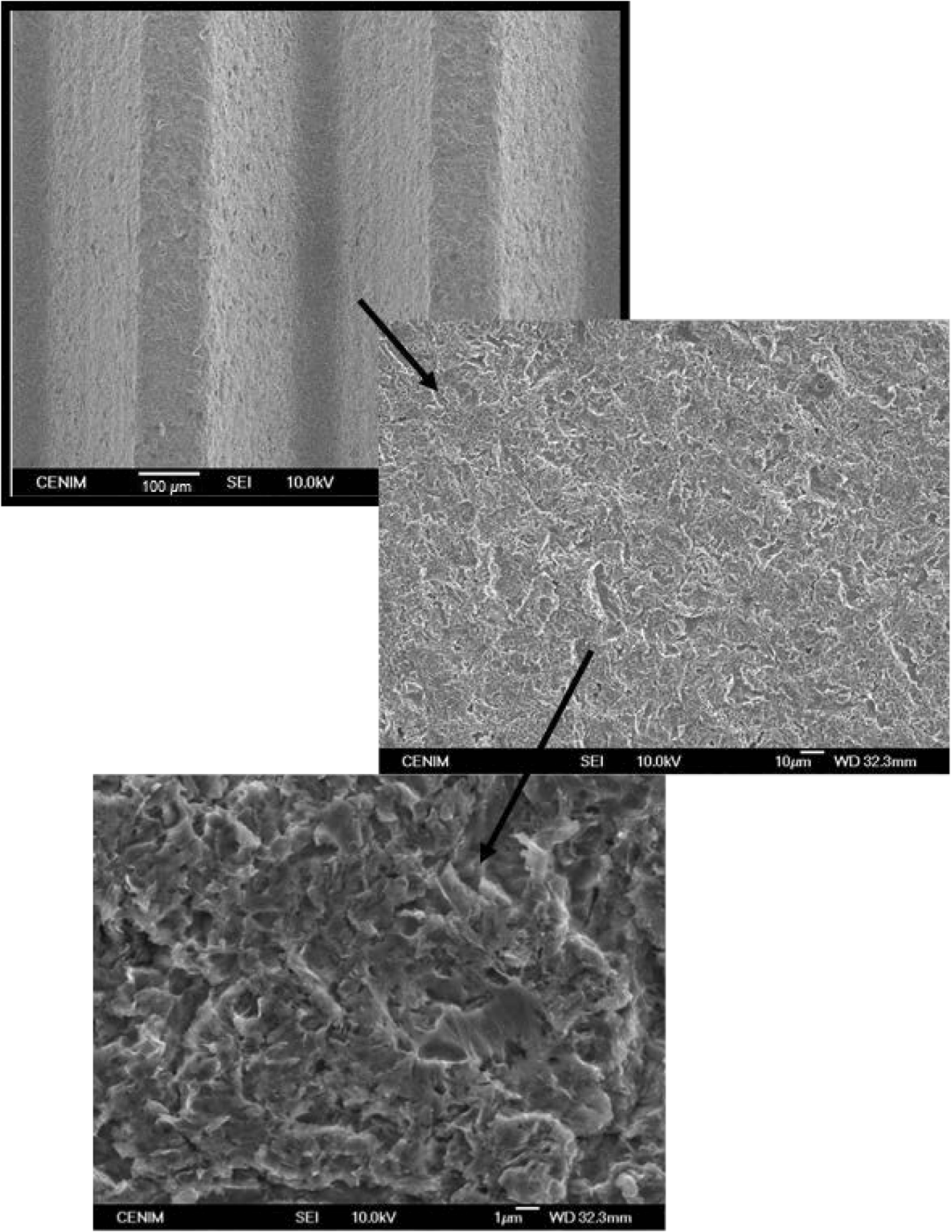
SEM image of the surface of control commercial Ti6Al4V dental implants

Figure 3 shows images at different magnifications by SEM of the typical threaded topography of the Ti6Al4V screws and the characteristic surface with cavities and holes heterogeneously dispersed due to the impingement of the particles used in the blasting process. The chemical analysis performed by EDX of a representative area (Table 1) shows the characteristics peaks of Ti, Al and V, together with some proportion of Ca and P. Ca and P came from the hydroxyapatite particles used in the blasting process.

**Table 1:** Chemical analysis by EDAX of the surface of Ti6Al4V commercial implants

Element	Atomic, %
Al	11.00
P	0.54
Ca	0.38
Ti	84.71
V	3.37

Due to this process, it is unavoidable that the blasting particles induces abrasive pollution on the surface, giving rise to a surface modification not only in the final roughness but also in the chemical composition, that influences the physicochemical properties of the blasted surfaces [25]. To minimize the drastic changes produced by of the blasting process, the posterior acid attack is usually used as a method to smooth titanium surfaces and also to eliminate the residual particles resulting from the blasting process [26]. The use of HCl posterior to the blasting process does not assure that residual hydroxyapatite particles remain incrusted in the surface of the implants. Nevertheless, given the chemical nature of the Ca-P compounds on the surface, its presence could be even considered as beneficial in the osseointegration process.

**Fig. 4:**
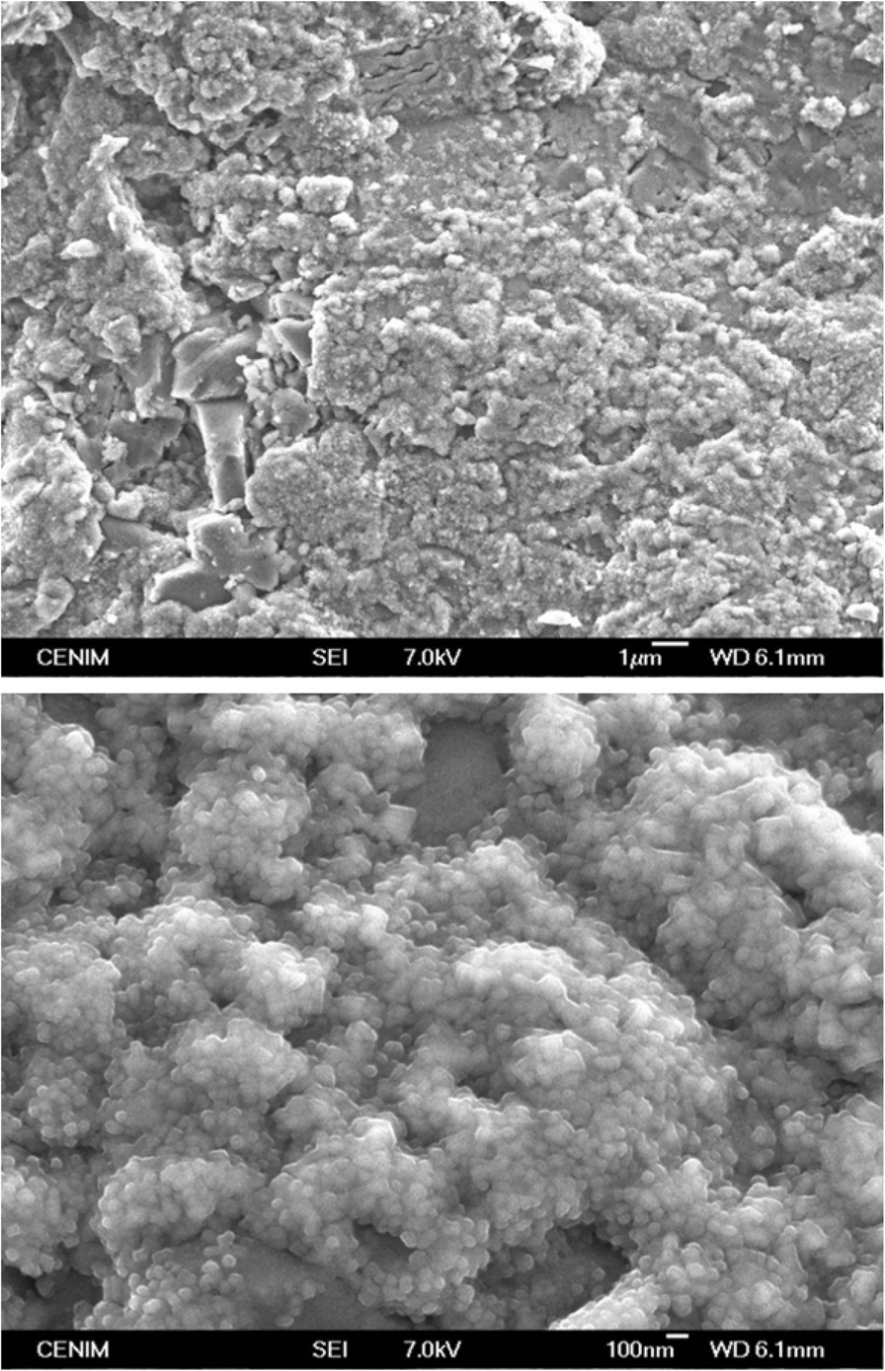
SEM image of the nanoroughness of the oxidized surfaces on control Ti6Al4V dental implants after 700 °C for 1 h

After the thermal treatment, an oxide scale is grown on the Ti6Al4V screws (Fig. 4). Barranco et al. [27] established that the increase in roughness (at microscale) of specimens due to the thermal treatment at 700 °C for 1 h was not significant. However, the nanoroughness of the surface can be increased about 140 nm due to this treatment [27] (compare Figs. 3 and 4). The thermal oxidation of the Ti6Al4V alloy after oxidation treatment at 700 °C for 1 h gives rise to a modified surface whose composition and crystalline order is changed. Previous XRD results of Ti6Al4V thermally treated at 700 °C for 1 h carried out by the authors and published in Billi et al. [28] revealed diffraction angles assigned to rutile, without any evidence of aluminium oxide. Confidence about the stability of the microstructure during the thermal oxidation treatments was provided in previous works by no significant differences in hardness from 329 to 320 HV0.5 after 700 °C for 1 h [4].

Table 2 shows the estimated mean values and standard deviations for the BMD in healthy (control) and osteoporotic (OVX) rabbits. The comparison of mean values established significant differences between control and OVX groups (*p* = 0.006). The BMD results show that the ovariectomy and hypocalcic diet described in (20) resulted being proper methods to have osteoporotic rabbits before the insertion of implants in the tibiae.

**Table 2:** Mean (grammes per square centimetre) and standard deviations of the bone mineral density (BMD) of tibiae and vertebral column in healthy (control) and osteoporotic (OVX) rabbits

Animal group	BMD (g cm^−2^) tibiae	BMD (g cm^−2^) vertebral column
Control	0.323 ± 0.014	0.285 ± 0.007
OVX	0.301 ± 0.016	0.271 ± 0.009

After insertion of the implants, BMD of above and below implant areas in tibiae with and without the local application of GH for each rabbit group (control and OVX) was measured at 15 and 30 days. As an example, means (grammes per square centimetre) and standard deviations in control and OVX rabbits after 15 days appear in Table 3.

**Table 3:** Means (grammes per square centimetre) and standard deviations of the bone mineral density (BMD) of above and below implant areas in tibiae with and without the local application of growth hormone (GH) in healthy (control) and osteoporotic (OVX) rabbits

Animal group		15d			
		Commercial		Modified	
		Without GH	With GH	Without GH	With GH
Control	Above	0.356 ± 0.017	0.368 ± 0.036	0.382 ± 0.006	0.351 ± 0.021
	Below	0.366 ± 0.008	0.372 ± 0.029	0.374 ± 0.031	0.366 ± 0.015
OVX	Above	0.308 ± 0.024	0.348 ± 0.019	0.357 ± 0.053	0.345 ± 0.027
	Below	0.369 ± 0.027	0.343 ± 0.011	0.363 ± 0.032	0.330 ± 0.024

BMD values indicate that there are no significant statistical differences either above or below the implant areas, in metaphysis and diaphysis regions, independently on the variables studied: type of implant (commercial or modified) and addition of GH local administration in the implantation site.

**Fig. 5:**
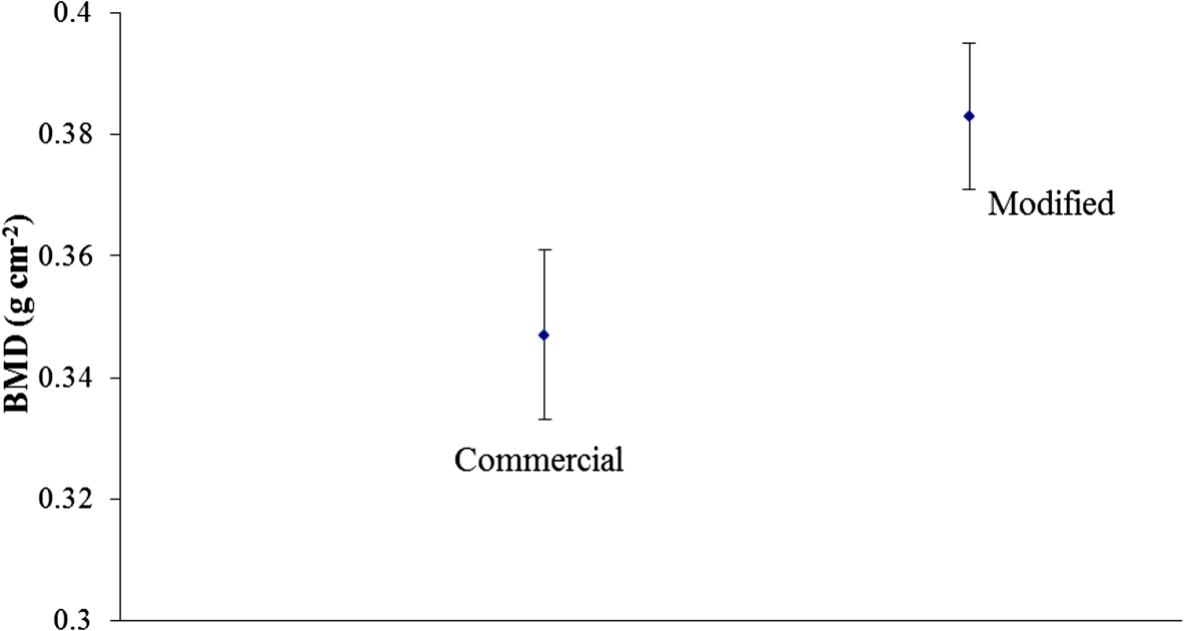
Bone mineral density (*BMD*) values in osteoporotic (OVX) rabbits after 30 days of implantation of both commercial and modified implants. Vertical lines represent standard deviations

In general, after 30 days, in OVX group, BMD results show that the modification of the surface by the thermal treatment gives rise to a significant better bone response (*p* = 0.016) (Fig. 5).

In agreement with the BMD results, the BIC values show no significant statistical differences independently on the local administration of GH in the implantation site and previous oxidation treatment of the implant.

**Fig. 6:**
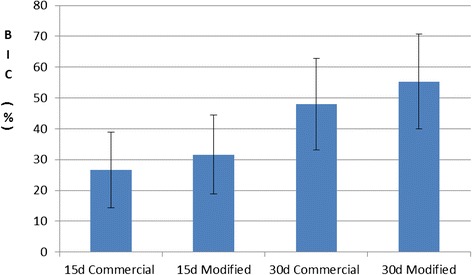
Bone to implant contact (BIC) values (%) for commercial and modified implants in osteoporotic (OVX) rabbits after 15 and 30 days of implantation. *Vertical lines* represent standard deviations

Figure 6 shows the results of BIC values of thermally treated and control implants in OVX rabbits (with and without GH) after 15 and 30 days. We found a small non-significant difference in BIC results in the osteoporotic group affecting the osseointegration response after 15 and 30 days of implantation period, mainly related to the previous thermal treatment applied on the implants. Nevertheless, all ANOVA values were higher than 0.05 so no significant differences were found at any condition at 95 % confidence level.

## Discussion

A good preparation of the implant surface in order to accommodate bone topographical features contributes to accelerate the assembly between the new bone formed and the metallic implant. Research has been consequently focused on the surface modifications of implants aimed to simulate the size of the proteins or cell membrane receptors. In vitro results about the influence of different micro-nanoscale roughness on the osteoblast differentiation are sometimes contradictory [29, 30]. This is probably due to the differences between the types of cells and the types of nanoscale surface modifications used in these experiments [31]. So, the definitive confirmation should be given by in vivo studies. In this work, the in vivo study about the use of oxidation treatments to modify the nanoroughness on the previous micro-scale roughness of the implant surface has proved to accelerate the osseointegration process.

The morphometric measurement of the BIC of osseointegrated implants is the standard procedure for the evaluation of bone formation on an implant surface. High BIC values are considered to be a prerequisite for implant stability, which clinically enables functional dental reconstruction. Studies evaluating new implant surfaces assess this parameter. In particular, differences in BIC between test and reference surfaces are statistically analysed to compare their osteogenic potential. Nevertheless, it is interesting to remark that the use of two (or even only one) histological sections per implant may strongly influence the determined BIC [32].

In our work, densitometric and morphometric studies point to a better bone response to the Ti6Al4V commercial implants thermally treated at 700 °C for 1 h (Figs. 5 and 6). The highest BIC value achieved for the thermally treated implant after 30 days of implantation (especially in the OVX rabbit group) could be explained by considering different features on the oxidized surface such as chemical composition and topographical properties. The chemical composition of the oxidized surface after heat treatment at 700 °C for 1 h, verified by XRD, is rutile [28], which is formed on the free-particle areas of the Ti6Al4V surface. Rutile scale has been proved to enhance the osteoblast response [4], improve the resistance to pitting corrosion [27] and decrease the ion release of the Ti6Al4V [5].

In addition, the morphology of the hard rutile scale grown on the implant surface increases nanoroughness (Fig. 4). Studies carried out by Webster et al. [29] have pointed out the importance of the surface roughness at the nanometre range in the connectivity to bone. It has been discussed that the increase of micro- and submicro-scale surface roughness, with feature sizes comparable to those of resorption pits of bone and cell dimensions, leads to enhanced osteoblasts differentiation and increased bone-to-implant contact in vivo [33].

In the specific case of osteoporotic rabbits, the results showed that the modification of the surface by the thermal treatment gives rise to a better bone response in BMD and BIC values at longer time of implantation, i.e., 30 days (Figs. 5 and 6). These results seem to indicate that the crystallography and the chemical composition given by the rutile enhance the interaction between osteoblasts and surface improving the bone regeneration around the Ti6Al4V implants.

With respect to local administration of GH to the osteoporotic rabbits, significant results have not been obtained, although a slight improvement was observed. In our study, densitometric results related to those animals in the GH treated group that were sacrificed after 15 days showed a tendency towards increased bone mineral density (BMD) that did not reach statistical significance. Our results emphasize the idea of the variability in the response to GH because, by itself, topical administration gives rise to very different results probably due to additional characteristics that affect to variables such as the host, the methodology of administration of GH or the implant itself. These results are in agreement with the authors’ previous data [11] and other authors [34]. For example, Calvo-Guirado et al. [14] found topical application of 4 IU of GH like biomimetic agent at the moment of implant placement has no significant effects on the BIC at 5 and 8 weeks, although bone formation and inter-thread bone values did increase significantly. However, controversial about the efficiency of the topical administration of GH is found in the literature. Munoz et al. [35] found that topical application of growth hormone GH and melatonin synergistically enhance new bone formation around titanium implants in early stages of healing.

During the first period of 15 days, the rabbit biokinetics is appropriate to initiate a formation of young bone, which can be confirmed or not, at 30 days. In this animal model, the bone formation can be initiated before 15 days, as has been seen in other studies, but it is very spars. Moreover, the first days guide us towards an evolution of the bone response that is confirmed at 15 days. It has been published in previous works [10] that the fast periosteal response found could be explained by direct action of GH on the pluripotential mesenchymal cells in the first steps of the repair process. The GH accelerated this process in what has been called the “impulse effect” stimulating osteoblasts, chondroblasts, and fibroblasts. However, the most authors have not statistically found a response to GH dependent dose [10, 12], asseveration that is in accordance with our data.

Systemic and local administration of growth factors accelerates bone regeneration [36] and promotes osteoinductive effects [37]. In fact, the systemic administration of GH has shown to accelerate fracture healing [38] at longer periods of implantation. The beneficial role of GH on the bone regeneration could be achieved at initial local administration of GH in the implantation site where oxidized Ti6Al4V implant is located and after a critical period of implantation time, systemic administration by means of small regular dose, according to [38].

## Conclusions

In summary, oxidation treatments of Ti6Al4V dental implants stimulate a better bone response at longer implantation times. The local application of GH on the implantation site showed no significant effect in the osseointegration process (from BMD and BIC measurements) of thermal and commercial Ti6Al4V dental implants during the first month of implantation.

## References

[1] Lomholt TC, Pantleon K, Somers MAJ (2011). In-vivo degradation mechanism of Ti-6Al-4V hip joints. Mater Sci Eng C.

[2] Wisbey A, Gregson PJ, Peter LM (1991). Effect of surface treatment on the dissolution of titanium-based implant materials. Biomaterials.

[3] Chen G, Wen X, Zhang N (1998). Corrosion resistance and ion dissolution of titanium with different surface microroughness. Biomed Mater Eng.

[4] Garcia-Alonso MC, Saldana L, Valles G (2003). In vitro corrosion behaviour and osteoblast response of thermally oxidised Ti6Al4V alloy. Biomaterials.

[5] Saldaña L, Barranco V, García-Alonso MC (2006). Concentration-dependent effects of titanium and aluminium ions released from thermally oxidized Ti6Al4V alloy on human osteoblasts. J Biomed Mater Res.

[6] Saldaña L, Vilaboa N, Vallés G (2005). Osteoblast response to thermally oxidized Ti6Al4V alloy. J Biomed Mater Res.

[7] Zhang L, Wu K, Song W (2015). Chitosan/siCkip-1 biofunctionalized titanium implant for improved osseointegration in the osteoporotic condition. Scientific Reports.

[8] Kammerer PW, Lehnert M, Al-Nawas B (2015). Osseoconductivity of a specific streptavidin-biotin-fibronectin surface coating of biotinylated titanium implants - a rabbit animal study. Clin Implant Dent Relat Res.

[9] Germanier Y, Tosatti S, Broggini N (2006). Enhanced bone apposition around biofunctionalized sandblasted and acid-etched titanium implant surfaces A histomorphometric study in miniature pigs. Clin Oral Impl Res.

[10] Tresguerres IF, Clemente C, Donado M (2002). Local administration of growth hormone enhances periimplant bone reaction in an osteoporotic rabbit model. An histologic, histomorphometric and densitometric study. Clin Oral Impl Res.

[11] Tresguerres IF, Blanco L, Clemente C (2003). Effects of local administration of growth hormone in periimplant bone: an experimental study with implants in rabbit tibiae. Int J Oral Maxillofac Implants.

[12] Tresguerres IF, Alobera MA, Baca R (2005). Histologic, morphometric, and densitometric study of peri-implant bone in rabbits with local administration of growth hormone. Int J Oral Maxillofac Implants.

[13] Lynch S, Buser D, Hernández RA (1991). Effects of the platelet derived growth factor insulin-like growth factor-I combination on bone regeneration around titanium dental implants. Results of a pilot study in beagle dogs. J Periodontol.

[14] Calvo-Guirado JL, Mate-Sanchez J, Delgado-Ruiz R (2011). Effects of growth hormone on initial bone formation around dental implants: a dog study. Clin Oral Impl Res.

[15] Oryan A, Alidadi S, MoshirI A (2014). Bone morphogenetic proteins: a powerful osteoinductive compound with non-negligible side effects and limitations. Biofactors.

[16] Becker W, Linch SE, Lekholm U (1992). A comparison of e-PTFE membranes alone or in combination with platelet-derived growth factors and insulin-like growth factor-I or demineralized freeze-dried bone in promoting bone formation around immediate extraction socket implants. Periodontol.

[17] Bak B, Andreassen TT (1991). The effect of growth hormone on fracture healing in old rats. Bone.

[18] Andreassen TT, Jorgensen PH, Flyvbjerg A (1995). Growth hormone stimulates bone formation and strength of cortical bone in aged rats. J Bone Miner Res.

[19] European Parliament and Council (1986). Council Directive 86/609/EEC of 24 November 1986 on the protection of animals used for experimental and other scientific purposes. Official J Eur Communities.

[20] Committee on Animal Nutrition; Board on Agriculture and Renewable Resources; National Research Council. Nutrient Requirements of Rabbit. 2nd rev. ed. Washington DC: National Academic of Sciences; 1977.

[21] Vidigal GM, Groisman M, Gregorio LH (2009). Osseointegration of titanium alloy and HA-coated implants in healthy and ovariectomized animals: a histomorphometric study. Clin Oral Impl Res.

[22] Martin-Monge E, Tresguerres IF, Blanco L (2011). Validation of an osteoporotic animal model for dental implant analyses: an in vivo densitometric study in rabbits. Int J Oral Maxillofac Implants.

[23] Donath K, Breuner G (1982). A method for the study of undecalcified bones and teeth with attached soft tissues. J Oral Pathol.

[24] Chappard D, Baslé MF, Legrand E (2011). New laboratory tools in the assessment of bone quality. Osteoporos Int.

[25] Barranco V, Escudero ML, Garcia-Alonso MC (2007). 3D, chemical and electrochemical characterization of blasted Ti6Al4V surfaces: Its influence on the corrosion behavior. Electrochim Acta.

[26] Diniz MG, Soares GA, Coelho MJ (2002). Surface topography modulates the oeteogenesis in human bone marrow cell cultures grown on titanium samples prepared by a combination of mechanical and acid treatments. J Mater Sci.

[27] Barranco V, Onofre E, Escudero ML (2010). Characterization of roughness and pitting corrosion of surfaces modified by blasting and thermal oxidation. Surf Coat Tech.

[28] Billi F, Onofre E, Ebramzadeh E (2012). Characterization of modified Ti6Al4V alloy after fretting–corrosion tests using near-field microscopy. Surf Coat Tech.

[29] Webster TJ, Ejiofor JU (2004). Increased osteoblasts adhesion on nanophase metals: Ti. Ti6Al4V and CoCrMo. Biomaterials.

[30] Cai KY, Bossert J, Jandt KD (2006). Does the nanometer scale topography of titanium influence protein adsorption and cell proliferation?. Colloids Surf B: Biointerfaces.

[31] Gittens RA, McLachlan T, Olivares-Navarrete R (2011). The effects of combined micron-submicron scale surface roughness and nanoscale features on cell proliferation and differentiation. Biomaterials.

[32] Bernhardt R, Kuhlisch E, Schulz MC (2012). Comparison of bone-implant contact and bone-implant volume between 2d-histological sections and 3D-SR-μCT slices. Eur Cells Mater.

[33] Cochran DL, Schenk RK, Lussi A (1998). Bone response to unloaded and loaded titanium implants with a sandblasted and acid-etched surface: a histometric study in the canine mandibule. J Biomed Mater Res.

[34] Lutz R, Srour S, Nonhoff J (2010). Biofunctionalization of titanium implants with a biomimetic active peptide (P-15) promotes early osseointegration. Clin Oral Imp Res.

[35] Muñoz F, López-Peña M, Miño N (2012). Topical application of melatonin and growth hormone accelerates bone healing around dental implants in dogs. Clin Oral Imp Res.

[36] Raschke MJ, Bail H, Windhagen HJ (1999). Recombinant growth hormone accelerates bone regenerate consolidation in distraction osteogenesis. Bone.

[37] Schmidmaier G, Wildemann B, Bail H (2001). Local application of growth factors (insulin-like growth factor-1 and transforming growth factor-β1) from a biodegradable poly(D, L-lactide) coating of osteosynthetic implants accelerates fracture healing in rats. Bone.

[38] Schmidmaier G, Wildemann B, Heeger J, et al. Improvement of fracture healing by systemic administration of growth hormone and local application of insulin-like growth factor-1 and transforming growth factor-β1. Bone. 2002;31:165–72. 10.1016/s8756-3282(02)00798-6 12110430

